# How to Best Exploit Immunotherapeutics in Advanced Gastric Cancer: Between Biomarkers and Novel Cell-Based Approaches

**DOI:** 10.3390/jcm10071412

**Published:** 2021-04-01

**Authors:** Michele Ghidini, Angelica Petrillo, Andrea Botticelli, Dario Trapani, Alessandro Parisi, Anna La Salvia, Elham Sajjadi, Roberto Piciotti, Nicola Fusco, Shelize Khakoo

**Affiliations:** 1 Medical Oncology Unit, Fondazione IRCCS Ca’ Granda Ospedale Maggiore Policlinico, 20122 Milan, Italy; 2 Medical Oncology Unit, Ospedale Del Mare, 80147 Naples, Italy; angelic.petrillo@gmail.com; 3 Department of Clinical and Molecular Medicine, Sapienza University, 00189 Rome, Italy; andrea.botticelli@uniroma1.it; 4 Medical Oncology (B), Policlinico Umberto I, 00161 Rome, Italy; 5 Division of Early Drug Development for innovative therapies, European Institute of Oncology, IRCCS, 20141 Milan, Italy; dario.trapani@ieo.it; 6 Department of Life, Health and Environmental Sciences, University of L’Aquila, 67100 L’Aquila, Italy; alexparis@hotmail.it; 7 Medical Oncology Unit, St. Salvatore Hospital, 67100 L’Aquila, Italy; 8 Department of Oncology, University Hospital 12 De Octubre, 28041 Madrid, Spain; alasalvi@ucm.es; 9 Division of Pathology, European Institute of Oncology, IRCCS, 20141 Milan, Italy; elham.sajjadi@ieo.it (E.S.); roberto.piciotti@unimi.it (R.P.); nicola.fusco@unimi.it (N.F.); 10 Department of Oncology and Hemato-Oncology, University of Milan, 20122 Milan, Italy; 11 Department of Medicine, Royal Marsden Hospital, London and Surrey, Sutton SM25PT, UK; shelize.khakoo@rmh.nhs.uk

**Keywords:** immune checkpoint inhibitors, gastric cancer, Epstein Barr Virus, tumor mutational burden, microsatellite instability, predictive biomarkers, CAR T cell therapy, vaccines

## Abstract

Despite extensive research efforts, advanced gastric cancer still has a dismal prognosis with conventional treatment options. Immune checkpoint inhibitors have revolutionized the treatment landscape for many solid tumors. Amongst gastric cancer subtypes, tumors with microsatellite instability and Epstein Barr Virus positive tumors provide the strongest rationale for responding to immunotherapy. Various predictive biomarkers such as mismatch repair status, programmed death ligand 1 expression, tumor mutational burden, assessment of tumor infiltrating lymphocytes and circulating biomarkers have been evaluated. However, results have been inconsistent due to different methodologies and thresholds used. Clinical implementation therefore remains a challenge. The role of immune checkpoint inhibitors in gastric cancer is emerging with data from monotherapy in the heavily pre-treated population already available and studies in earlier disease settings with different combinatorial approaches in progress. Immune checkpoint inhibitor combinations with chemotherapy (CT), anti-angiogenics, tyrosine kinase inhibitors, anti-Her2 directed therapy, poly (ADP-ribose) polymerase inhibitors or dual checkpoint inhibitor strategies are being explored. Moreover, novel strategies including vaccines and CAR T cell therapy are also being trialed. Here we provide an update on predictive biomarkers for response to immunotherapy with an overview of their strengths and limitations. We discuss clinical trials that have been reported and trials in progress whilst providing an account of future steps needed to improve outcome in this lethal disease.

## 1. Introduction

### Overview of Gastric Cancer Classification and Relevance for Immunotherapy

Gastric cancer (GC) is a leading global cause of morbidity and mortality [1]. In 2020, over a million people were diagnosed with GC (representing almost 6% of all cancer diagnoses), and nearly 800,000 patients died due to this disease (representing 8.2% of all cancer deaths) [2]. Worldwide, GC is particularly prevalent in East Asia and central/Eastern Europe.

The Lauren classification, published in 1965, differentiates gastric adenocarcinoma into two distinct types, termed the intestinal and diffuse subtypes [3]. The intestinal type is most common, present in over half of the patients and characterized by microscopic glandular structures, with infiltrating capacity of the mesenchymal tissues [4]. The diffuse subtype accounts for a third of cases and is characterized by poor differentiation and poorly cohesive malignant cells with invasive capacity [5]. In general, the intestinal type is associated with exogenous risk factors such as Helicobacter pylori, while the diffuse subtype encompasses a hereditary familial pattern related to germline pathogenetic mutations of the E-cadherin (CDH1) and αE-catenin (CTNNA1) genes [6]. While these subtypes of GC are associated with different carcinogenesis mechanisms and disease biology, this classification, along with the subsequent World Health Organization classification of GC, has not translated into distinct subtype-driven treatment strategies [7,8]. More recently, following comprehensive molecular profiling, The Cancer Genome Atlas (TCGA) defined four distinct subtypes of gastric cancer: Epstein-Barr virus (EBV) positive, microsatellite unstable tumors (MSI), genomically stable tumors (GS) and tumors with chromosomal instability (CIN) [9]. Significant overlap was seen between the histologically determined Lauren’s diffuse variant and the molecular GS TCGA subtype [10]. Interestingly, certain molecular subtypes were most commonly detected in specific anatomic locations with EBV positive tumors more likely to be in the gastric fundus or body and CIN tumors in the cardia [9]. Although the molecular classification of gastric cancer has not directly changed clinical practice, it has provided an important platform to identify novel molecular targets and pave the way for innovative clinical trial design with the incorporation of biomarker enrichment stratification strategies. EBV-positive and MSI tumors are associated with signatures suggestive of an immune responsive profile [11]. A hyper-mutated DNA phenotype is defined as 20.5 mutations/Mb in GC and is a phenotype typical of most MSI tumors [12]. The MSI high (MSI-H) phenotype is most commonly related to epigenetic silencing of the mismatch repair gene, MLH1, rather than germline mutation (i.e., Lynch syndrome) [13]. The presence of a higher number of somatic mutations has been associated with a better prognosis [14] and an increased susceptibility to immune-activating antineoplastic treatments [15]. Currently, patients with MSI gastric cancer can benefit from established immunotherapy approaches with anti-programmed death-1 (anti-PD-1) immune-checkpoint inhibitors [16]. Rather than a hypermutated phenotype, EBV-positive tumors (accounting for 9% of GC) have a profile favoring immunotherapy in view of their high expression of membrane immune-checkpoint molecules such as programmed death ligand-1 (PD-L1) and 2. Key molecular features of EBV-positive tumors include the expression of virus-associated antigens (e.g., nuclear antigen 1, latent membrane protein 2A), the extensive methylation of viral and host genome and the epigenetic regulation of specific cytosine-phosphatidyl-guanosine (CpG) DNA islands through methylation mechanisms [17]. The pattern of DNA methylation of CpG has been associated with anti-tumor immune-activation, with predictive and prognostic significance [18,19]. Therefore, MSI and EBV-positive tumors have been proposed as chief candidates for immunotherapy trials, though not exclusively, for their intrinsic immune-mediated biology [11]. The advent of immunotherapy in oncology has in fact been embraced in most if not all tumor types and disease settings [20]. The identification of an immune-signature or predictive factors of immune-response in patients with GC have been identified as a research priority given that it is a tumor type associated with poor prognosis when diagnosed at an advanced stage and any benefit derived from chemotherapy (CT) is very limited [21]. While advancements in the development of pharmacotherapies have improved overall survival (OS) and quality of life, the low proportion of patients alive after two years from the diagnosis of metastatic disease remains a cause for concern [22,23].

The strategies implemented to enhance the immune response against tumors, including GC, aim to re-orient the immune-system response, by dampening the suppressive regulatory molecules and enhancing a stimulating milieu [24]. This strategy has been pursued by developing a number of immune-checkpoint inhibitors [e.g., PD-1, cytotoxic T-lymphocyte antigen-4 (CTLA-4)], a class of molecules capable of acting on several immune cells and (re-)activating an effective antineoplastic response [25]. This strategy is particularly beneficial in tumors exerting immune-activating signatures and/or recognized by the immune-system as foreign, and therefore regulated by the immune-response [26].

Another therapeutic approach is based on the bioengineering of immune-competent cells against specific tumor- associated antigens [27]. The principal expression of this approach is represented by the Chimeric Antigen Receptor T-cells (CAR-T) constructs. CAR-T are genetically engineered T-cells designed to direct the specific immune-response against tumor- antigens, thereby inducing an artificial acquired antineoplastic immune response, through cytotoxic activity. Though still widely experimental in solid neoplasms, the clinical implementation of CAR-T cells for hematological malignancies has paved a new way of cancer immunotherapy, due to the durable responses seen in some cases, the different patterns of response observed [28] as well as the specific safety profile which needs to be considered and the structural efforts required to build and deliver cell-based treatments [29].

Here we review the clinical and translational landscape of the determinants of response to various immunotherapy agents in patients with GC, by elucidating the key findings from clinical trials and describing established and proposed predictive biomarkers throughout ongoing clinical studies incorporating immunotherapy.

## 2. Biomarkers of Response to Immunotherapy in Gastric Cancer

**Figure 1:**
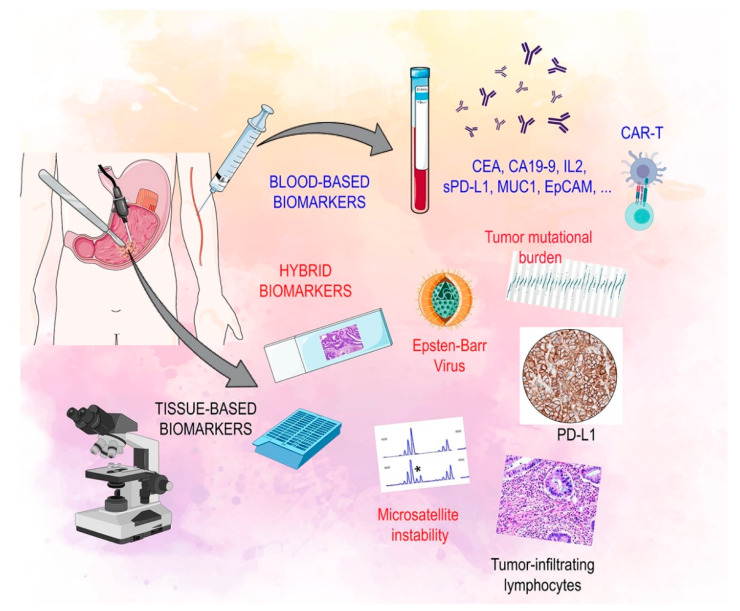
Biomarkers of response to immunotherapy: soluble, tissue based and hybrid. Legend: CAR-T: chimeric antigen receptor-T cell, CA 19.9: carbohydrate antigen 19.9, CEA: carcinoembryonic antigen, EPCAM: epithelial cell adhesion molecule, IL2: interleukin 2, MUC1: mucin 1, cell surface associated, PD-L1: programmed death-ligand 1, s PD-L1: serum programmed death-ligand.

The characterization of immune-related biomarkers is becoming increasingly important in the multi-modality treatment of advanced GC (Figure 1) [30].

### 2.1. Tissue Based Biomarkers

Currently, the most studied biomarkers include mismatch repair (MMR) status assessment, MSI identification, PD-L1 expression, tumor-infiltrating lymphocytes (TILs) assessment, and tumor mutational burden (TMB) quantification. However, there is currently a gap in knowledge regarding the reliability of these tests for clinical use in GC. MMR deficiency (dMMR) and/or MSI has been reported in approximately 14% and 22% of GCs, respectively [31,32]. The MMR system is able to identify and counteract unpaired DNA bases in order to preserve genome stability [33,34,35]. Alterations in this system, due to dMMR, are associated with the accumulation of alterations in microsatellite regions, resulting in variable degrees of MSI that are commonly defined as “low” (MSI-L) and “high” (MSI-H) [35]. MMR and MSI screening is recommended as a useful tool at all stages of GC to refine treatments and determine patient prognosis [36]. In GC, MSI is more common with older age, female sex, distal stomach location, lower number of lymph-node metastases and is associated with an overall better prognosis [36,37,38]. According to a meta-analysis which included 1556 resectable GC patients, MSI-H patients had longer five-year OS and disease-free survival (DFS) compared to patients with microsatellite stable tumors (OS, 77.5% vs. 59.3%; DFS, 71.8% vs. 52.3%) [14,39]. Evidence suggests that dMMR is more likely to activate an immune response and lead to the increased presence of TILs, and PD-L1 upregulation in GC [40,41,42,43]. In particular, PD-L1 is expressed on the surface of neoplastic cells in 15–70% of GC [37], with increased expression being associated with non-metastatic cancer tissue [44], well differentiated tumors [44] and improved OS (median OS not reached vs. 40 months; *p* = 0.008) [45] although its association with a favorable OS has not always been consistent [37,46]. The immunohistochemical expression of PD-L1 protein can be scored using the combined positive score (CPS), where CPS > 1 is considered positive [47]. There appears to be an association between PD-L1+ GC and MSI-H or EBV positive tumors [48]. The evaluation of PD-L1 CPS on formalin-fixed paraffin-embedded tumor tissue samples has been proposed as a method to select patients for immune-checkpoint inhibition [49]. High PD-L1 CPS score has been associated with a high density of CD3+/CD8+ TILs. Interestingly, PD-L1 negative tumors with high-density CD3+ and CD8+ cells had a good prognosis [46]. A meta-analysis on various TIL subtypes in GC has shown that high levels of CD8+, CD3+, and CD4+ T cell infiltration is associated with better OS. Additionally, a high density of forkhead box P3 (FOXP3) positive cells within the tumor does not appear to be a negative prognostic indicator [50]. TILs are gathering increasing importance as a prognostic biomarker in GC [51]. Regarding TIL assessment in GC, only the stromal count (= % area occupied by mononuclear inflammatory cells over the total stromal area)—stained by hematoxylin and eosin—has been suggested for evaluation, due to a lack of prognostic significance for intra-tumoral TILs [52]. However, this finding requires further validation [52,53].

In the KEYNOTE 059 study, PD-L1 expression as a potential biomarker of response to pembrolizumab in advanced and refractory GC patients was evaluated and demonstrated a higher overall response rate (ORR) in PD-L1+ compared to the PD-L1 negative tumors (15.5%, CPS ≥1 vs. 6.4%, CPS < 1). However, PD-L1 negative tumors displayed greater complete response rate (CR) (2.8% vs. 2.0%) [39,54]. Such findings prompted the need for defining and utilizing further biomarkers of response to immunotherapy. Tumor mutational burden (TMB), dMMR/MSI, TILs and EBV have been broadly explored as the main molecular determinants of immunotherapy response in GC. Of note, TMB (i.e., the number of somatic mutations derived from next-generation sequencing techniques) has been correlated with higher levels of neoantigen expression, and subsequently increased immune responses [55]. High TMB has been suggested in 3% and 5% of patients with esophageal and stomach cancer, respectively (>20 mut/Mb) [30]. Recently, the food and drug administration (FDA) approved the FoundationOneCDx assay (Foundation Medicine, Inc., Cambridge, MA, USA) as a companion diagnostic (CDx) for treatment with pembrolizumab in unresectable or metastatic TMB-high solid tumors (≥10 mut/Mb) [56]. Findings in GC also show an improved OS in TMB-High tumors treated with immunotherapy, compared to those with lower TMB levels: 80% 2-year survival for TMB-high vs. 12% for TMB-low, *p* = 0.03 [57] and median OS = 16.8 vs. 6.62 months, *p* = 0.058 [58]. Yet, TMB as a potential biomarker of response to immunotherapy is challenged by the lack of harmonized sequencing panels as well as lack of clearly defined cut-offs for implementation in clinical practice [36].

MSI-H tumors have also been associated with a good response to immunotherapy [15,59,60]. Based on these findings, the FDA approved pembrolizumab for the treatment of MSI-H tumors that had progressed following prior treatment, irrespective of tumor site [61]. In GC, studies have also demonstrated that dMMR and MSI-H tumors generally have a favorable response to immune-checkpoint blockade [36,59]. A multi cohort study of pembrolizumab monotherapy in advanced GC showed that MSI-H tumors had greater ORR (57.1%) compared to MSS patients (9%) and also a significant disease control rate (DCR) of 71.4% was achieved [30,36]. These results were supported by findings from the phase III, KEYNOTE-062 clinical trial which are discussed in the clinical trial section of this review [62]. MMR status is commonly assessed by immunohistochemistry although the lack of CDx tests and tumor-specific guidelines is seen as a disadvantage [35]. However, unlike in other types of tumors, a high correlation between MMR immunohistochemistry and MSI testing in gastro-esophageal cancer is generally observed [36]. Hence, the PCR-based Bethesda panel, consisting of two mononucleotide repeats and three dinucleotide repeats, and NGS are employed in MSI evaluation [63].

EBV+ tumors are associated with a response to immunotherapy and this appears to be independent of TMB and MSI status [64]. According to a multi-factorial genomic biomarker analysis in GC patients administered pembrolizumab, EBV positivity, MSI and PD-L1 expression are associated with improved ORR (100%, 85.7%, and 50%, respectively) [65]. EBV+ GC appears to be relatively immunogenic. This results in increased infiltration with immune cells and also increased PD-L1 and PD-L2 gene expression [65]. Further investigation into biomarkers of response to immunotherapy in advanced GC/gastro-esophageal cancer is warranted. A summary of the most commonly studied biomarkers along with their strengths and limitations is provided in Table 1.

### 2.2. Circulating Biomarkers

Circulating molecules and their role in predicting response to immunotherapy is a topic of great interest and is an area that still has not been studied in depth. These soluble factors can be released from both tumor cells and immune cells and may provide a simple method to evaluate the dynamic behavior of the immune system in cancer patients during treatment and avoid the need for invasive procedures [66]. Much effort is being spent on identifying primary responders to immunotherapy at a relatively early treatment timepoint. Circulating biomarkers, some of which are currently used in clinical practice, such as pepsinogen, carcinoembryonic antigen (CEA), carbohydrate antigen 19-9 (CA19-9) and soluble IL-2, are not accurate enough to predict prognosis in GC [67,68,69].

Recent evidence has shown that patients with GC have higher serum soluble PD-L1 (sPD-L1) concentrations than healthy controls. Moreover, both elevated tissue PD-L1 and serum sPD-L1 were independent prognostic factors for poor OS and poor DFS in GC patients who underwent surgery [70,71,72]. Lymphocyte activation gene 3 (LAG3) is a checkpoint receptor localized on activated T cell surfaces and NK cells. The soluble variant, in turn, can have a regulatory function on immune cells [73,74]. Its role has been investigated in GC patients. High sLAG-3 expression is associated with a better prognosis in GC and its expression was positively correlated with IL-12 and IFN-γ production in GC patients. In a recent in vivo experiment sLAG3 was shown to be able to promote the activation of CD8 T cells and the production of INF and IL-12, resulting in tumor growth inhibition [75].

While the prognostic value of soluble checkpoints is under investigation in several solid tumors, the question that remains to be answered is whether soluble checkpoints can predict response to treatment. Given that the immune system is a key factor involved in the response to treatments such as immunotherapy and CT, there is a clear rationale to suggest that such soluble markers could be biomarkers of response to treatment [76]. A study including 11 patients with NSCLC and 9 patients with GC treated with an anti-PD1 agent showed that pre-treatment levels of sPD-L1 were not associated with OS in these patients. However, reduction in plasma sPD-L1 levels was significantly associated with tumor response after four cycles of treatment [77]. A study including 68 patients with metastatic GC eligible for first line CT analyzed baseline level of sPDL1 and the dynamic changes during therapy. Patients with low levels of sPD-L1 at diagnosis showed a better OS and PFS than patients with a high sPDL1. Patients whose sPDL1 increased after the first cycle of CT showed worse PFS and OS. This result suggests that soluble checkpoints may be the ideal method of studying the immune system as an extremely dynamic entity allowing real-time, non-invasive monitoring during cancer treatment [78]. Takahashi et al. confirmed in their study that high serum levels of sPD-L1 correlated with worse OS in patients with metastatic GC treated with first-line CT [79]. These data suggest the possibility of individualizing the therapeutic choice based on the immunological profile, thereby leading to promising new combination strategies in the near future.

Immunotherapeutics in solid tumors is constantly evolving due to the introduction of new technologies to manipulate the patient’s immune system to attack cancer cells. Tumor antigen vaccines are currently being studied in several solid tumors. They are created from cancer cells’ pure tumor antigens [80]. The antitumor activity of tumor peptide vaccines, such as G17DT, vascular endothelial growth factor receptor (VEGFR) and OTSGC-A24, have been investigated in GC patients. G17DT is a vaccine able to promote an immune response against gastrin, a hormone involved in carcinogenesis and progression in GC [81,82,83]. A phase II/III study (NCT00042510) reported that G17DT is able to induce efficient anti-gastrin antibody production and is able to inhibit tumor proliferation and progression [84]. A multi-center study showed that the combination of G17DT and platinum-5FU CT prolonged the median time-to-progression and median survival time for patients with unresectable cancer of the stomach or gastroesophageal junction, compared to platinum-5FU CT alone. Therefore, the FDA approved the fast track designation for the vaccine G17DT in February 2003 [85]. Another peptide vaccine involving the use of VEGFR 1 and 2, receptors of the VEGF angiogenic factor, has been investigated. In a phase I/II study, the administration of the VEGFR1/2 peptide vaccine in combination with CT induced a cytotoxic T cell response. In the 82% of patients with a cytotoxic T lymphocyte response to VEGFR2-169 peptide, time to progression and OS were significantly prolonged compared to those without such a response [86]. Such findings are encouraging, although it should be noted that only 22 patients were included. A phase I/Ib study (NCT01227772) evaluated OTSGC-A24, which is thought to be able to target several specific tumor antigens, such as forkhead box M1, DEP domain containing 1, kinesin family member 20A, URLC10 and VEGFR1. Although the treatment was well tolerated, no radiological responses were observed [83].

An innovative immunotherapeutic strategy uses adoptive T cell therapy to overcome the immune-evasion mechanisms mediated by cancer cells. T lymphocytes are removed from patients and modified in vitro in order to activate specific immune cells. Then, the modified activated T cells are administered to patients, thereby eliciting a tumor response against cancer [87]. Chimeric antigen receptor-T (CAR-T) cell therapy was shown to be effective in hematologic disease and it is actually under investigation in several solid tumors [88,89].

In GC, several antigens, including human epidermal growth factor receptor 2 (HER2), carcinoembryonic antigen (CEA), mucin 1 (MUC1) and epithelial cell adhesion molecule (EpCAM), have been used as targets for CAR-T. The anti-HER2 CAR-modified T cell was evaluated in many pre-clinical studies [90]. Clinical studies are now evaluating it in GC patients (NCT02713984, NCT01935843, NCT00889954). CEA-specific CAR-T cells were confirmed to be active in pre-clinical studies in mice with GC. Since then, a clinical trial is ongoing (NCT02349724) to define the correct dose and safety profile [91,92]. MUC1 and EpCAM are transmembrane glycoproteins expressed in different solid tumors, but in GC they are markers of aggressive disease. Clinical Phase I/II trials (NCT02617134, NCT02725125) are evaluating EpCAM and MUC1 modified CAR-T in solid tumors expressing these targets [93].

## 3. Immunotherapy: From Landmark Trials to Clinical Practice and Future Perspectives

Over the last decade, the safety and efficacy of immunotherapy has been investigated in clinical trials in GC patients, initially in the advanced disease setting and more recently in the earlier disease setting.

### 3.1. Non-Metastatic Disease

Most immune checkpoint inhibitor trials in the earlier disease setting are ongoing (Table 2). The use of immune checkpoint inhibitors- alone or in association with CT is not currently considered standard of care [94].

In the context of neoadjuvant and perioperative treatments, the phase III Keynote 585 trial (NCT03221426) is evaluating the efficacy of pembrolizumab plus CT versus CT alone [95]. The CT arm was initially cisplatin plus capecitabine or cisplatin plus fluorouracil. Following the favorable results of the FLOT-4 study, the trial protocol was amended to enable the inclusion of the FLOT CT regimen comprising fluorouracil, docetaxel and oxaliplatin as a safety cohort [96]. The primary endpoints are OS, event free survival (EFS) and the rate of pathological complete response (pCR). It is important to note that PD-L1 status is not being used for patient selection, although an exploratory endpoint assessing efficacy by PD-L1 expression is planned.

In the adjuvant setting, the initial results from the phase III Checkmate 577 trial were recently presented [97]. This trial (NCT02743494) assessed the safety and efficacy of nivolumab versus placebo as adjuvant treatment in 794 patients with stage II and III esophageal (squamous tumors and adenocarcinomas) and esophagogastric junctional adenocarcinoma (GEJA) who had received neoadjuvant treatment followed by surgery. Patients were not selected for PD-L1 status. Nivolumab significantly prolonged DFS (22.4 versus 11 months, Hazard Ratio (HR): 0.69; *p* = 0.0003) with a good safety profile (grade 3–4 adverse events: 13% versus 6% in the placebo arm). Whilst these initial results are promising, the full publication is awaited in order to analyze the data in more detail. Additionally, it should be noted that the trial included both esophageal and GEJ cancers as well as squamous tumors and adenocarcinomas. Therefore, it could be argued that these tumor types should be assessed separately in dedicated clinical trials to better understand clinical applicability. For additional details regarding ongoing trials in these settings, see Table 2.

### 3.2. Metastatic Disease: 1st Line Treatment

Evidence for the role of immune checkpoint inhibitors in first-line treatment of metastatic GC is very recent, arising during the last few months. In this regard, pembrolizumab, nivolumab and avelumab are the main agents that have been investigated (Table 3).

The phase III Keynote 062 trial was a study with a complex design, including both superiority and non-inferiority comparisons. In fact, in the superiority part, the trial evaluated the safety and efficacy of pembrolizumab plus standard CT (cisplatin plus 5-fluorouracil/capecitabine) versus CT alone in first-line treatment of metastatic GC/GEJA patients with epidermal growth factor receptor 2 (HER-2) negative status. Additionally, the trial included a third arm, evaluating the non-inferiority of pembrolizumab as a single agent treatment and compared it to standard CT [62]. Therefore, 763 patients (Asian and non-Asian) were randomized 1:1:1 to one of the three arms. The central assessment of PD-L1 was mandatory at screening and only patients with a PD-L1 ≥ 1 tumor according to the CPS score were randomized. After a median follow up of 29.4 months, single agent pembrolizumab was found to be non-inferior to the control arm (median OS: 10.6 versus 11 months; HR: 0.91; 99.2% Confidence Interval (CI): 0.69–1.18; non-inferiority margin: 1.2) with a trend of superiority for patients with PD-L1 CPS ≥ 10 (median OS: 17.4 versus 10.8 months, HR: 0.69). However, this last analysis was not planned. The survival rates at 12 and 24 months were 46.9% and 26.5% in the experimental single agent arm versus 45.6% and 19.2% in the control arm. Nevertheless, the trial did not improve OS in the combination arm, both for patients with PD-L1 CPS ≥ 1 (median OS: 12.5 versus 11.1 months; HR: 0.85, 95% CI: 0.70–1.03, *p*: 0.05) and PD-L1 CPS ≥ 10 (median OS: 12.3 versus 10.8 months; HR: 0.85; 95%CI: 0.62–1.17; *p*: 0.16). Likewise, the superiority in PFS was not met for the experimental arm (median PFS: 6.9 versus 6.4 months; HR: 0.84; 95%CI: 0.70–1.02; *p*: 0.04). Of note, patients with MSI-H benefited the most from pembrolizumab both for patients with PDL-1 CPS ≥1 (median OS: not reached (NR) versus 8.5 months in the control arm, HR: 0.29) and PDL-1 CPS ≥ 10 (median OS: NR versus 13.6 months). The survival benefit was maintained in this subgroup also in the combination arm (pembrolizumab plus CT: HR: 0.37).

Recently, the preliminary results of the phase III Checkmate 649 [98] and ATTRACTION-4 trial [99] were presented at the ESMO Congress 2020. The Checkmate 649 trial (NCT02872116) randomized untreated metastatic GC patients to three arms: nivolumab plus ipilimumab, standard CT (Folfox or Xelox), standard CT plus nivolumab [98]. Patients were enrolled regardless of PD-L1 status and HER-2 testing was not mandatory, although patients with known HER-2 positive tumors were excluded. The preliminary results only reported the analysis for the combination arms (1581 patients) and, among those patients, the data focused on those with PDL-1 CPS ≥ 5 (955 patients, 60%). In this population, the experimental arm (nivolumab plus CT) was associated with improved survival benefit when compared to CT alone (median OS: 14.4 versus 11.1 months, respectively, HR: 0.71, *p* < 0.0001; median PFS: 7.7 versus 6.1 months, HR: 0.68; *p*: < 0.0001). However, the benefit was also confirmed in the entire population- including PD-L1 negative tumors- (median OS: 13.8 versus 11.6 months, respectively, HR: 0.8, *p*: 0.0002) as well as in the PD-L1 CPS ≥ 1 subgroup (median OS: 14 versus 11.3 months, respectively, HR: 0.77, *p*: 0.0001). The safety profile was acceptable and the rate of grade 3–4 adverse events for the experimental versus control arm was 59% versus 44%, respectively. Of note, the trial included 75% non-Asian patients. Therefore, the combination of Folfox/Xelox plus nivolumab seems to be very promising in first-line treatment of metastatic disease in GC. However, the full publication is awaited in order to better understand the biological mechanisms that underpin the positive results and to understand how this combination could be used in clinical practice.

The phase III ATTRACTION-4 trial (NCT02746796) randomized 724 Asian patients to receive CT alone (Xelox or oxaliplatin plus S-1) or with nivolumab as first-line treatment for HER-2 negative metastatic GC, regardless of PDL-1 status [99]. After a follow up of 11.6 months, the addition of nivolumab improved PFS when compared with the control arm (median PFS: 10.5 versus 8.3 months, respectively; HR: 0.68, *p*: 0.0007). However, with a median follow-up of 26.6 months, OS was not significantly different in the two arms (median OS: 17.5 versus 17.2 months, HR 0.90; 95% CI: 0.75–1.08; *p*: 0.257), whereas the benefit in PFS and overall response rate (ORR) were confirmed (ORR: 57.5 versus 47.8%; *p*: 0.0088). Therefore, the Checkmate 649 and ATTRACTION-4 trials provide the first evidence for the efficacy of nivolumab in this setting, even if in different populations. However, the final results and publication from the ATTRACTION-4 trial, are also awaited.

The phase III Keynote-859 [106] and Keynote-811 [107] are currently ongoing in this setting (Table 2). Keynote-859 (NCT03675737) is randomizing untreated metastatic HER-2 negative GC patients to receive standard CT (cisplatin plus 5-fluorouracil/Xelox, investigator choice) alone or with pembrolizumab as first-line treatment [106]. The trial includes patients regardless of PD-L1 status; however, assessment of PD-L1 status is mandatory. The Keynote-811 trial (NCT03615326) is investigating the role of pembrolizumab in first-line treatment for HER-2 positive metastatic GC [107]. The trial was based on the promising results of the phase Ib/II trial PANACEA trial [108] in breast cancer and in the following phase II study in esophageal/GEJA/GC [100]. This latter phase II study was an open-label, non-randomized, single-arm trial that showed promising activity and a good safety profile by using pembrolizumab in addition to standard CT (Folfox/Xelox plus trastuzumab or cisplatin plus 5-fluorouracil/capecitabine plus trastuzumab- according to investigator’s choice) in 37 HER-2 positive tumors (5% Asian patients), regardless of PD-L1 status. Of the patients, 70% were alive at six-months and free from relapse (primary endpoint), median PFS and median OS were 13 and 27.3 months, respectively. Of note, ORR was 100%, 17% had a complete response and 74% a partial response. These initial results are of significant interest as they show the potential for a new treatment option in patients with HER-2 positive tumors if confirmed in a larger phase III study. The results of the randomized phase III Keynote-811 trial which includes patients with the same characteristics are therefore eagerly awaited.

Maintenance treatment with immunotherapy after first-line therapy has also been investigated. The phase III Javelin 100 trial assessed the efficacy and safety of using an immune checkpoint inhibitor (avelumab) in this setting [101]. The trial randomized 805 metastatic HER-2 negative GC/GEJA patients who demonstrated a response to first line CT (Folfox/Xelox) to receive either CT (continuation of the ongoing treatment) or avelumab. The trial included Asian patients (20%); patients were not selected by PD-L1 status, although a subgroup analysis for PD-L1 CPS ≥ 1 was pre-planned. The trial failed to show an improvement in OS with avelumab in this setting (median OS: 10.4 versus 10.9 months, HR: 0.91, 95% CI: 0.74–1.11; *p*: 0.1779). These results were also confirmed in the PD-L1 positive population (median OS: 16.2 versus 17.7 months, HR: 1.13; 95% CI: 0.57–2.23, *p*: 0.6352). However, in this trial, the small MSI-H subgroup of patients appeared to benefit from immunotherapy (HR: 0.27; 95% CI: 0.06–1.25).

The results from studies using immune checkpoint inhibitors in first-line treatment for metastatic GC are promising. However, it is not yet entirely clear which patients benefit the most from immunotherapy due to the lack of reliable, validated predictive biomarkers to guide the treatment choice for patients with GC [109]. Therefore, the search for new biomarkers as well as a better understanding of the molecular mechanisms underlying the response to immunotherapy is urgently needed. Immunotherapy does not currently represent standard of care in the first line metastatic setting in GC and its use is restricted by local authorities.

### 3.3. Metastatic Disease: Second Line Treatment and Beyond

Following progression with a first-line platinum- and fluoropyrimidine-based CT regimen [110], the VEGFR2 human monoclonal antibody (mAb) ramucirumab is a standard of care in the second-line setting. Ramucirumab monotherapy improved OS when compared to best supportive care (BSC) (5.2 vs. 3.8 months, HR = 0.77, *p* = 0.047) and also in association with paclitaxel when compared to paclitaxel alone (9.6 vs. 7.4 months, HR = 0.80, *p* = 0.017) in two randomized phase III trials (REGARD and RAINBOW, respectively) [111,112]. While efficacy and safety of ramucirumab were confirmed in “real-life” populations [113,114,115], the randomized phase III TAGS trial confirmed the OS benefit of the cytotoxic oral drug trifluridine/tipiracil over placebo (5.7 vs. 3.6 months, HR = 0.69, *p* = 0.0005) as a third-line regimen in a global population [116].

The role of immunotherapy was first demonstrated when monotherapy with PD-1 inhibitors (nivolumab and pembrolizumab) showed significant efficacy in later lines of treatment [54,103,117] (Table 3). In the phase III ATTRACTION-02 trial, nivolumab significantly prolonged OS compared to placebo (5.2 vs. 4.1 months, HR = 0.63, *p* < 0.0001) in patients progressed or intolerant to at least two previous lines of treatment, with a 3-year OS rate of 5.6% and 1.9%, respectively. Therefore, only a small proportion of patients achieved durable clinical benefit from nivolumab. Notably, PD-L1 status did not identify patients likely to benefit from nivolumab. Toxicity profile included mild to moderate diarrhea, fatigue, pruritus, and rash. However, longer OS was observed in patients experiencing immune checkpoint inhibitor (ICI)-related AEs compared to those who did not (2-year OS of 20% and 0%, respectively). These findings resulted in the approval of nivolumab in third- or later-line in Japan, Taiwan and South Korea [103]. Similar data were obtained in Western populations [117] even if phase III data are lacking.

The human anti-PD1 pembrolizumab was first tested in the phase II KEYNOTE-059 trial and obtained higher ORR (15.5% vs. 6.4%) and longer duration of response (DOR, 16.3 vs. 6.9 months) in PD-L1-positive (CPS ≥ 1%) rather than in PD-L1-negative mGC as a third- or later-line treatment. These results led to FDA approval of pembrolizumab as a third- or later line of treatment for CPS ≥ 1% mGC patients in the USA [54].

The positive results of the ATTRACTION-02 trial were not replicated in two phase III trials testing the efficacy of PD1/PD-L1 inhibitors compared to CT. In the JAVELIN Gastric 300 trial, the PD-L1 inhibitor avelumab did not improve survival over standard CT (mOS: 4.6 vs. 5.0 months, *p* = 0.81, respectively) in third-line treatment, irrespective of PD-L1 status (TPS ≥ 1%) [105].

In the KEYNOTE-061 trial, 592 mGC patients progressed on a first-line platinum- and fluoropyrimidine-based CT were randomized to receive pembrolizumab or paclitaxel as second-line. Primary endpoints were OS and PFS in patients with PD-L1 CPS ≥ 1, with significance threshold for OS set at *p* = 0.0135 (one-sided). Pembrolizumab failed to improve outcome in terms of OS (mOS: 9.1 vs. 8.3 months, HR = 0.82, *p* = 0.042) and PFS (mPFS: 1.5 vs. 4.1 months) when compared to paclitaxel alone [102]. One of the main limitations of this trial was the control arm of paclitaxel without ramucirumab, which is considered standard of care in the second-line setting of mGC. In an updated 2-year analysis of the KEYNOTE-061 trial, a trend towards improved OS in PD-L1 CPS ≥ 1 patients in favor of pembrolizumab was shown. Moreover, a higher benefit from pembrolizumab over paclitaxel in terms of OS, ORR and DOR was described in subgroups of patients with performance status 0, CPS ≥ 10% and MSI-high [118].

As multiple immune checkpoint pathways modulate antitumor response, combining PD1/PD-L1 inhibitors with other ICIs is a potential strategy to overcome resistance. For example, the immune checkpoint molecule CTLA-4 suppresses T-cell proliferation early in the immune response, whereas PD-1 acts in a later phase of T-cell suppression [119].

The phase I-II CheckMate-032 trial tested nivolumab alone or in combination with the inhibitor of cytotoxic T-lymphocyte associated protein-4 (CTLA-4) ipilimumab in 160 pretreated Western mGC patients, reaching an ORR of 12% and 24% and G3–4 AEs of 17 and 47%, respectively. These results were obtained regardless of PD-L1 status [104].

The tumor microenvironment (TME) is an integral part of cancer and includes a variety of immune and non-immune cell types and factors playing a pivotal role in driving an inflammatory, immunosuppressive and pro-angiogenic intra-tumoral environment [120]. Tumor-associated macrophages (TAMs) play a role in cancer microenvironment, they can affect inhibitory and growth cancer cell processes depending on stage, tissue type, and host microbiota [121]. Furthermore, TAMs can impact on the antitumor effects of CT and radiotherapy and contribute to intrinsic/acquired resistance to PD-1 inhibitors [122]. Interestingly, these cells can be reduced by inhibiting the colony-stimulating factor-1 (CSF-1)/receptor pathway. The association of the CSF-1 inhibitor lacnotuzumab and the PD-1 inhibitor spartalizumab is under investigation in a phase II trial enrolling pre-treated patients (Table 2). Within the TME, tumor neo-vascularization promoted by tumor-induced angiogenic factors can lead to an imbalance between immunosuppressive cells such as regulatory T cells (Treg) and TAMs, and anti-tumor CD8+ cytotoxic T-lymphocytes (CTLs), causing tumor progression, invasion and angiogenesis [120]. Anti-angiogenic agents may restore the anti-tumor immune activity by disrupting the VEGF/VEGFR axis in the TME [122]. On the other hand, the association of immunotherapy and CT might be of benefit by improving immunogenicity and restoring balance within the TME [120]. This strategy is currently under investigation, safety and activity data from combinations treatments such as paclitaxel and ramucirumab with avelumab (RAP: NCT03966118) or pembrolizumab (SEQUEL: NCT04069273) (Table 2) are awaited.

The multi-targeted tyrosine kinase inhibitor (TKI) regorafenib enhances antitumor immunity through macrophage modulation [123]. In the Japanese EPOC1603 phase Ib trial, the combination of regorafenib and nivolumab showed anti-tumor activity (ORR 44%, mPFS 5.8 months) [124]. The multi-targeted TKI lenvatinib was evaluated together with pembrolizumab in the Japanese EPOC1706 phase II trial, showing promising activity (ORR 69%) [125].

Genomic instability derives from deficient DNA damage response. Poly (ADP-ribose) polymerase (PARP) inhibitors (PARPi) alter the ability to repair DNA damage; their effect is more pronounced in tumors with pre-existing defects in DNA repair (such as MSI/dMMR tumors). Unrepaired DNA damage secondary to PARPi treatment was reported to activate immune pathways and PD-L1 expression on tumor cells, which could in turn increase sensitivity to ICIs [126]. Phase II trials combining ICIs and PARPi with or without VEGFR inhibitors or CT are ongoing (Table 2).

The HER-2 inhibitor trastuzumab has immune mechanisms of action involving innate and adaptative immunity through antibody-dependent cellular cytotoxicity, upregulation of PD-L1 and promotion of immune infiltration [127,128]. In the global phase I-II CP-MGAH22-05 trial, the association of the novel anti-HER2 mAb margetuximab and pembrolizumab provided positive results in terms of safety and efficacy (ORR 18%, DCR 53%) in 95 pre-treated HER2-positive mGC patients [129].

The composition of the gut microbiome has emerged as a key factor affecting the peripheral immune system in the context of cancer. Moreover, gut microbiota might affect the efficacy of immune checkpoint inhibitors in various cancers [127]. The DELIVER trial (JACCRO GC-08, UMIN000030850) aims to investigate the role of immune-related biomarkers (gut microbiome, genetic polymorphisms, gene expression, and the metabolome in plasma) in patients treated with nivolumab.

## 4. Discussion

In recent years, immunotherapy has revolutionized cancer care. Due to its efficacy, its long-lasting effect and its relative favorable safety profile, this innovative approach has changed the natural history of different types of tumors, such as lung cancer, head and neck and urological malignancies. For patients with mGC the prognosis disappointingly remains dismal. The standard therapies (chemotherapy, trastuzumab or ramucirumab) have limited impact on patient outcomes, and median survival ranges from four months with BSC only, to 12 months with chemotherapy [22]. Therefore, improving the knowledge of the GC molecular landscape as well as developing targeted therapies may serve as a promising approach in the treatment of GC patients. To date, several studies have been carried out and many are ongoing, aimed to define the magnitude of benefit and the role for immunotherapy, as monotherapy and combined with chemotherapy, targeted agents, and other immunotherapies, in GC. In this context, a huge variety of biomarkers have shown promising results, particularly MSI, PD-L1 and TMB, as well as soluble biomarkers, including sPD-L1, sLAG-3, circulating tumor DNA (ctDNA), exosomes, cytokines, cancer-testis antigens (CTA) [130] and metal chelators, and finally the microbiome [131]. Unfortunately, none of these biomarkers has been validated for use in clinical practice, so far.

Therefore, discovering reliable predictive biomarkers of response to immunotherapy in GC represents a critical unmet need to personalize treatment and improve survival. Despite the success achieved with ICIs for the treatment of other solid tumors, the results in the treatment of GC are uncertain, although the benefit appears to be more pronounced in patients with PD-L1+ expression, MSI-H or dMMR tumors [132]. Consequently, nowadays, the approved indications for immunotherapy in mGC are limited to second or subsequent lines of therapy. However, surgery remains the only curative option in GC and immunotherapy may play an important role even in the neoadjuvant and adjuvant setting. Most trials of immune checkpoint blockade in the earlier disease are ongoing, such as the phase III Keynote 585 trial in the neoadjuvant setting [95] and the Checkmate 577 trial in adjuvant [97]. Combining immunotherapy and chemotherapy might lead to improved tumor immunogenicity, and, in this way, improve immunotherapy efficacy. The rationale to combine an immunosuppressive drug, such as chemotherapy, with agents that act to modulate immune regulatory mechanisms to boost the immune response against cancer cells, is potentially challenging. In mGC the addition of chemotherapy to immunotherapy by increasing TMB with platinum agents could be especially interesting [133].

Several trials are further investigating the activity of the association between ICIs and different chemotherapy agents, as first- or second-line treatment for mGC. Of these, the results of combining paclitaxel and ramucirumab with avelumab (RAP: NCT03966118) or pembrolizumab (SEQUEL: NCT04069273) are eagerly awaited.

Additionally, numerous clinical trials evaluating the combination of immunotherapy with targeted agents (anti-angiogenic agents, PARPi and anti-HER2 mAb) are generating much excitement. In this context, there is a strong rationale to combine ICIs with anti-angiogenic drugs. Preclinical evidence has demonstrated that normalizing the tumor vasculature enhances immunotherapy activity. Notably, evidence suggests that enhanced immune stimulation improves tumor vascular normalization [134]. In mGC encouraging results have been observed for this strategy (i.e., combining nivolumab plus ramucirumab [135] or regorafenib) [124]. However, the level of evidence for these combinations is still limited, thereby hampering their approval for clinical use.

## 5. Conclusions

In conclusion, despite the efforts made, GC remains a highly lethal cancer and the magnitude of benefit from immunotherapy for these patients is still under debate. A critical open question regarding patient selection for treatment with immunotherapy and the optimal sequence of where it should be used in the treatment paradigm remains. Nowadays, a plethora of potentially useful predictive biomarkers has been investigated, but unfortunately their clinical use is still limited. Additionally, the results achieved with immunotherapy in the metastatic setting are encouraging, but not completely satisfactory. Further studies are urgently needed to deepen the molecular knowledge of the GC milieu. It is hoped that this will eventually lead to a more clearly defined algorithm of key criteria to select candidates likely to obtain the most benefit from immunotherapy.

## Figures and Tables

**Table 1:** Biomarkers for immunotherapy: detection methods, strengths and limitations.

Biomarker	Method and Interpretation	Clinical Value	Clinical Setting	Strengths	Limitations
**PD-L1**	IHC 22C3 (CDx): positive if CPS ≥ 1	Predictive (pembrolizumab); prognostic (poor OS)	FDA: advanced or metastatic GC/GEJC treated with ≥2 lines of therapy	Standardized (CDx), reliable	Relatively expensive if CDx, poor inter-observer reproducibility, high intra-tumor heterogeneity
**MMR**	IHC for MLH1, MSH2, MSH6, and PMS2: deficient if lack of expression in ≥1 biomarker	Predictive (pembrolizumab); prognostic (improved OS)	Tissue/site-agnostic: unresectable or metastatic dMMR GC/GEJC progressed following prior treatment	Reliable, cost-effective, short turn-around times	No CDx and interpretation guidelines, no data on intra-tumor heterogeneity
**MSI**	FoundationOne (CDx); MSI-H by PCR or NGS: hyper-variability ≥2 Bethesda (BAT-25, BAT-26, D2S123, D5S346 and D17S250) or Promega (BAT-25, BAT-26, MONO-27, NR-21 and NR-24) loci	Predictive (pembrolizumab and other ICI) prognostic (improved OS); Validated with tumor specific guidelines	Tissue/site-agnostic: unresectable or metastatic MSI-H GC/GEJC progressed following prior treatment	CDx available, cost-effective (PCR or NGS if high volume)	Expensive (CDx or NGS if low volume), externalized analysis (CDx), no tumor-specific guidelines
**TILs**	sTILs on HE-stained sections; modified from International TILs Working Group guidelines for breast carcinoma (% of the tumor stromal area containing infiltrating mononuclear inflammatory cells)	Predictive for immunotherapies (emerging); Prognostic (improved RFS).	Not performed in clinical practice	Cost-effective	Controversial clinical value
**TMB**	FoundationOne (CDx); NGS: TMB-H if >17 mut/MB; SNVs counting by Oncomine Tumor Mutation Load Assay	Predictive for ICB; Prognostic (enhanced ORR and PFS); associated with clinical response to ICI	Not performed in clinical practice	CDx available	Expensive, externalized analysis (CDx), no guidelines, controversial clinical value
**EBV**	cobas EBV (CDx); EBV-encoded RNA ISH	Prognostic (improved OS and decrease of metastases recurrence); associated with amplification and/or overexpression of PD-L1 and PD-L2 in GC; high density of immune cell infiltration; alterations in the PIK3CA gene	Diagnostic/subtyping	Standardized and cost-effective	Not available in all centers

Legend: CDx: companion diagnostic, CPS:combined positive score, EBV: Ebstein-Barr virus, GC: gastric cancer, GEJC: gastro-esophageal junctional cancer, HE: hematoxylin and eosin, ICB: immune checkpoint blockade, ICI: immune checkpoint inhibitor, IHC: immunohistochemistry, ISH: in situ hybridization, MMR: mismatch repair, MSI: microsatellite instability, OS: overall survival, PCR: polymerase chain reaction, PFS: progression-free survival, RFS: relapse-free survival, SNVs: single nucleotide variants, TILs: tumor infiltrating lymphocytes, TMB: tumor mutational burden, TMB-H: tumor mutational burden-high.

**Table 2:** List of major ongoing phase I-III trials with immune checkpoint inhibitors in gastric cancer.

Study Name (NCT Number)	Country	Phase	Line	N	Drugs (Target)	Selected Population	Study Intervention I Experimental Arm/Control Arm or II Experimental Arm	Primary Endpoint
Non-metastatic gastric cancer								
Keynote-585 (NCT03221426)	Global	III	Perioperative	NA	Pembrolizumab (PD-1)	All comers	fluorouracil/capecitabine plus cisplatin or FLOT +/- pembrolizumab	OS EFS pCR
IMAGINE (NCT04062656)	Western	rII	Perioperative	NA	Nivolumab (PD-1) Ipilimumab (CTLA-4) Relatlimab (LAG-3)	All comers	FLOT Nivolumab Nivolumab + ipilimumab Nivolumab + relatlimab	pCR
NCT04354662	Asian	II	Perioperative	NA	Toripalimab (PD-1)	All comers	FLOT + toripalimab	DFS
ICONIC (NCT03399071)	Western	II	Perioperative	NA	Avelumab (PD-L1)	All comers	FLOT + avelumab	pCR
NCT03878472	Asian	II	Neoadjuvant	NA	SHR1210 (PD-1)	All comers	SHR1210 SHR1210 + Apatinib SHR1210 + Apatinib + S-1 SHR1210 + Apatinib+ S-1 + oxaliplatin	pRR
Checkmate-577 (NCT02743494)	Global	III	Adjuvant	794	Nivolumab (PD-1)	All comers	Nivolumab versus placebo after neoadjuvant chemoradiotherapy and surgery	DFS
EORTC VESTIGE (NCT03443856)	Western	rII	Adjuvant	NA	Nivolumab (PD-1) Ipilimumab (CTLA-4)	All comers	Nivolumab + ipilimumab versus FLOT after neoadjuvant FLOT and surgery	DFS
Metastatic gastric cancer								
Keynote-859 (NCT03675737)	Global	III	1°	NA	Pembrolizumab (PD-1)	HER-2 negative	cisplatin plus 5-fluorouracil/Xelox +/- pembrolizumab	OS PFS
Keynote-811 (NCT03615326)	Global	III	1°	NA	Pembrolizumab (PD-1)	HER-2 positive	cisplatin plus 5-fluorouracil/Xelox/Folfox/S-1 oxaliplatin + trastuzumab +/- pembrolizumab	PFS OS
APICAL-GE (NCT04278222)	Asian	II	1°	NA	Toripalimab (PD-1)	MSS	Anlotinib Plus Toripalimab	ORR
NCT04202484	Asian	II	1°	NA	Toripalimab (PD-1)	HER-2 negative	Toripalimab combined with oxaliplatin and Tegafur, Gimeracil and Oteracil Porassium Capsules	ORR
SHR-1210-III-316 (NCT04342910)	China	III	2°	550	Camrelizumab (PD-1) Apatinib (VEGFR2)	All comers	Camrelizumab + apatinib paclitaxel or irinotecan	OS
NCT04435652	Asia	II-III	2°	492	QL1604 (PD-1)	HER-2 negative	QL1604 + nab-paclitaxel followed by QL1604 maintenance paclitaxel alone	ORR, safety, OS
SEQUEL (NCT04069273)	USA	rII	≥2°	58	Pembrolizumab (PD-1) Ramucirumab (VEGFR2)	All comers	Paclitaxel + ramucirumab + pembrolizumab (patient-tailored algorithm) Paclitaxel + ramucirumab + pembrolizumab	ORR
DURIGAST (PRODIGE59-FFCD1707) (NCT03959293)	France	rII	2°	105	Durvalumab (PD-L1) Tremelimumab (CTLA-4)	All comers	FOLFIRI + durvalumab + tremelimumab FOLFIRI + durvalumab	PFS
ESR-15-11655 (NCT03579784)	Korea	II	2°	40	Durvalumab (PD-1) Olaparib (PARP)	All comers	Paclitaxel + olaparib + durvalumab	DCR
NCC2070 (NCT04140318)	China	II	2°	60	Sintilimab (PD-1)	All comers	Nab-paclitaxel + sintilimab	ORR
ASGARD (NCT04089657)	China	II	≥3°	40	Sintilimab (PD-1) Apatinib (VEGFR2)	All comers	Apatinib + sintilimab	DCR
RiME (NCT03995017)	USA	II	2°–3°	61	Nivolumab (PD-1) Rucaparib (PARP) Ramucirumab (VEGFR2)	All comers	Rucaparib + ramucirumab + nivolumab Rucaparib + ramucirumab	ORR
RAP (AIO-STO-0218) (NCT03966118)	Germany	II	2°	59	Avelumab (PD-1) Ramucirumab (VEGFR2)	All comers	Paclitaxel + ramucirumab + avelumab	OS
WaKING (NCT04166721)	UK	II	≥2°	52	Atezolizumab (PD-L1) DKN-01 (DKK1)	MSS/pMMR	Atezolizumab + DKN-01	Safety, ORR
NCT03694977	Korea	II	>2°	30	Lacnotuzumab (CSF-1) Spartalizumab (PD-1)	All comers	Lacnotuzumab + Spartalizumab	Biomarker analysis
NCT04592211	Korea	I-II	2°	71	Pembrolizumab (PD-1) Olaparib (PARP)	HRR/MSS	Pembrolizumab + olaparib + paclitaxel	PFS DLT
NCT04209686	Australia, USA	II	2°	36	Pembrolizumab (PD-1) Olaparib (PARP)	All comers	Pembrolizumab + olaparib + paclitaxel	OS
da VINci (NCT03784040)	Asia	Ib	>2°	40	OTSGC-A24 (cancer vaccine) Nivolumab (PD-1) Ipilimumab (CTLA-4)	All comers	OTSGC-A24 + nivolumab OTSGC-A24 + nivolumab + ipilimumab	Safety, ORR

Legend: N: patient number; r: randomized; CPS: combined positive score for PD-L1 status; MSS/pMMR: microsatellite stable/mismatch repair proficient; DLT: dose-limiting toxicity; DCR: disease control rate; DOR: duration of response; ORR: objective response rate; OS: overall survival; EFS: event free survival; pCR: pathological complete response; PFS: progression-free survival; NA: not applicable; pRR: pathological remission rate.

**Table 3:** List of major completed phase II-III trials with immune checkpoint inhibitors in metastatic gastric cancer.

Study Name [Reference]	Agents (Target)	Country	Phase	Line	PD-L1 Status	Treatment Arms	N	Primary Endpoints	OS	PFS	RR (%)
Keynote-062 [62]	Pembrolizumab (PD-1)	Global	III	1°	CPS ≥ 1%	cisplatin + 5-fluorouracil/capecitabine (CT)	250	OS, PFS	Non-inferiority: 10.6 (I) vs. 11 (CT) Superiority: 12.5 (CT + I) vs. 11.1 (CT)	Superiority: 6.9 (CT + I) vs. 6.4 (CT)	48.6 (CT + I) 37.2 (CT)
						cisplatin + 5-fluorouracil /capecitabine + pembrolizumab (CT + I)	257				
						pembrolizumab (I)	256				
Checkmate-649 (preliminary results) [98]	Nivolumab (PD-1) Ipilimumab (CTLA-4)	Global	III	1°	Unselected	nivolumab + ipilimumab		OS, PFS		CPS ≥ 5%: 7.7 6.1	NR
						Xelox/Folfox	482		14		
						Xelox/Folfox + nivolumab	473		11.3		
Attraction-4 (preliminary results) [99]	Nivolumab (PD-1)	Asian	III	1°	Unselected	Nivolumab + S-1 oxaliplatin/Xelox	362	PFS, OS	17.5	10.5	NR
						S-1 oxaliplatin/Xelox	362		17.2	8.3	
Janjigian et al. [100]	Pembrolizumab (PD-1)	Global	II	1°	Unselected	Xelox/Folfox/cisplatin plus 5-fluorouracil+ trastuzumab+ pembrolizumab	37	PFS at 6 months	27.3	13	100
Javelin Gastric 100 [101]	Avelumab (PD-L1)	Global	III	1°mantainance	Unselected	Avelumab	249	OS	10.4	3.2	13.3
						Folfox/Xelox	250		10.9	4.4	14.4
Keynote-061 [102]	Pembrolizumab (PD-1)	Global	III	2°	CPS ≥ 1%	Pembrolizumab	196	PFS, OS	9.1	1.5	16
						Paclitaxel	199		8.3	4.1	14
Keynote-059 (cohort 1) [54]	Pembrolizumab (PD-1)	Global	II	≥3°	Unselected (57.1% CPS ≥ 1%)	Pembrolizumab	259	RR	5.6	2	11.6
Attraction-02 (ONO-4538-12) [103]	Nivolumab (PD-1)	Asian	III	≥3°	Unselected	Nivolumab	330	OS	7.5	1.6	11
						Placebo	163		5.1	1.5	20
Checkmate-032 [104]	Nivolumab (PD-1) Ipilimumab (CTLA-4)	Western	I-II	≥3°	Unselected	Nivolumab	59	RR	6.2	1.4	12
						Nivolumab1/Ipilimumab3 *	49		6.9	1.6	24
						Nivolumab3/Ipilimumab1 **	52		4.8	1.6	8
Javelin Gastric 300 [105]	Avelumab (PD-L1)	Global	III	3°	TPS ≥ 1%	Avelumab	272	OS	4.6	1.4	4.6
						Physician’s choice ^‡^	133		5	2.7	5

* Nivolumab 1 mg/kg plus ipilimumab 3 mg/kg every 3 weeks. ** Nivolumab 3 mg/kg plus ipilimumab 1 mg/kg every 3 weeks. ^‡^ Paclitaxel 80 mg/m^2^ on days 1, 8 and 15 or irinotecan 150 mg/m^2^ on days 1 and 15, each of a 4-week treatment cycle. Legend: N: patient number; NR: not reported; CPS: PD-L1 combined positive score; TPS: PD-L1 tumor proportion score; PD-1: programmed cell death protein-1; CTLA-4: Cytotoxic T-Lymphocyte Antigen 4; PD-L1: programmed cell death protein-ligand 1; OS: overall survival (months); PFS: progression-free survival (months); RR: response rate; NR: not reported.

## Data Availability

Not applicable.
